# Using multimodal imaging to improve the diagnostic accuracy and confidence in distinguishing non-arteritic anterior ischemic optic neuropathy from optic disc drusen

**DOI:** 10.3389/fneur.2026.1653402

**Published:** 2026-02-10

**Authors:** Min Young Kim, Anas Alkhabaz, Miaomiao Yu, Rishita Pujari, Yaping Joyce Liao

**Affiliations:** 1 Department of Ophthalmology, Stanford University, Palo Alto, CA, United States; 2 Department of Foundational Medical Studies, Oakland University William Beaumont School of Medicine, Rochester, MI, United States; 3 Department of Neurology, Stanford University, Palo Alto, CA, United States

**Keywords:** anterior ischemic optic neuropathy, multimodal imaging, ophthalmic imaging, optic disc drusen, optic disc edema, optic nerve head elevation, papilledema, pseudoedema

## Abstract

**Introduction:**

Optic nerve head elevation (ONHE) is a common diagnostic challenge in the general eye clinic. When caused by optic disc edema (ODE), ONHE may signal a neuro-ophthalmic emergency requiring urgent and invasive evaluation, whereas pseudoedema typically does not. This study evaluated whether multimodal oph¬thalmic imaging improves diagnostic accuracy and confidence in distinguishing nonarteritic anterior ischemic optic neuropathy (NAION), used as a model of true ODE, from optic disc drusen (ODD), a common cause of pseudoedema.

**Methods:**

We prospectively collected multimodal ophthalmic images using fundus color, near-infrared reflectance (NIR), fundus autofluorescence (FAF), and spectral-domain optical coherence tomography (OCT) optic nerve head and retinal nerve fiber layer (RNFL) analysis from 98 subjects (149 eyes: 60 NAION, 59 ODD, 30 controls). After a two-hour training session with a neuro-ophthalmologist, two masked medical trainees (a senior medical student and a recent medical gradu¬ate) independently reviewed single-, dual-, or multimodal image sets using a 0–5 confidence-weighted scale to assess for NAION, ODD, and control. Diagnostic accuracy was calculated using a weighted scoring system that penalized uncer¬tainty and misclassification. Confidence levels were categorized as high (defi¬nite), medium (likely), or low (maybe).

**Results:**

Among single imaging modalities, NAION diagnostic accuracy was highest with RNFL (83.2%) and color fundus imaging (80.9%), and lowest with FAF (65.4%). Combining color + RNFL improved accu¬racy to 88.1%. For ODD, FAF alone yielded the highest accuracy of 82.3%. The diagnostic accuracy of control images was consistently high across all single modalities (84.2 to 93.8%). Multimodal imaging produced the highest accuracy overall (NAION 93.4%, ODD 90.5%, controls 99.5%). The highest improvement using multimodal imaging was in NAION diagnostic confidence, which improved from 3 to 37% with single modality to 28 to 82% with multimodal imaging. We used mixed ANOVA and chi-square tests to evaluate diagnostic accuracy and grader confidence across modalities.

**Discussion:**

Brief training combined with multimodal imaging significantly improved diagnostic accuracy and confidence in differentiating NAION, ODD, and healthy controls. These findings support the potential clinical value of multimodal imaging in urgent-care settings where rapid and reliable evaluation of ONHE is essential.

## 1 Introduction

Optic nerve head elevation (ONHE) is a common diagnostic challenge in ophthalmology and optometry clinics. Optic disc edema (ODE) is a common cause of ONHE and may signify a possible neuro-ophthalmic emergency requiring urgent brain imaging, lumbar puncture, and other workup. A study in emergency rooms has shown that only 14% of patients presenting with headache, acute focal neurologic deficit, elevated blood pressure, or acute visual changes, were evaluated with direct ophthalmoscope (1). Among patients without previous ocular findings, its diagnostic accuracy in detecting optic nerve was 0% (1). Moreover, misdiagnosis of pseudoedema and ODE is common, with one study finding that 40% of pseudoedema initially being referred for ODE (2).

The most common concern in the setting of ODE is papilledema, or ODE due to elevated intracranial pressure—a common neuro-ophthalmic emergency. Papilledema may be related to the presence of an intracranial process such as tumor, infection, hemorrhage and is associated with high risk of vision loss and possible mortality. Other common differential diagnoses for ODE include anterior ischemic optic neuropathy (AION), the most common cause of acute optic neuropathy in adults older than 50 years of age (3, 4). AION can be due to giant cell arteritis, a medium to large vessel vasculitis associated with high morbidity and mortality, or more commonly is nonarteritic (NAION) with an incidence rate of 2.3–10.2 per 100,000 annually (3–5). NAION typically presents with acute painless vision loss and optic nerve edema due to hypoperfusion (6) with 15% of patients having second eye involvement within 5 years (7). The diagnostic challenge for those not familiar with different causes of ONHE is in distinguishing these causes associated with ODE with the many causes of ONHE without ODE, which do not necessarily need urgent assessment and are not associated with high risk of morbidity or mortality.

Common causes of ONHE without ODE include optic disc drusen (ODD), which is present in about 2% of the general population (8). The anatomical position of ODD can elevate the optic nerve head, mimicking ODE (3, 9). Moreover, ODD is the most common independent risk factor for younger onset NAION due to optic nerve crowding (10, 11), with studies showing greater than 50% of subjects younger than 50 years having ODD-associated NAION (ODD-AION) (12–14). As such, having ODD means there is an increased risk of true ODE (10), and papilledema vs. pseudopapilledema due to ODD is one of the most common referrals to the pediatric and adult neuro-ophthalmology clinic. Finally, remodeling of the optic nerve head for many reasons such as myopic degeneration, glaucoma, or optic nerve hypoplasia can increase the difficulty of accurately diagnosing ODE or pseudoedema.

The diagnostic challenge of ODE or not has inspired multiple studies, including some using non-mydriatic cameras at emergency rooms (15) and others using artificial intelligence approaches to assess the diagnostic accuracy of ODE (16–18). These emerging approaches may 1 day improve the diagnosis and management of patients with ONHE. However, the most important immediate need is to identify the best way we can educate our general providers and trainees to better diagnose ODE in acute presentation. There is currently no systematic curriculum for teaching the review of ophthalmic imaging in medical training.

In this study, we assess how different ophthalmic imaging modalities—color fundus photography, fundus autofluorescence (FAF), near-infrared reflectance (NIR), and retinal nerve fiber layer (RNFL) analysis—individually and in combination influence the accuracy and confidence of distinguishing optic disc edema (ODE) from pseudoedema. Using multimodal images from patients with NAION, ODD, and healthy controls, we aim to characterize the diagnostic value each modality contributes to the evaluation of optic nerve head elevation.

## 2 Materials and methods

### 2.1 Subjects and data collection

Images were prospectively collected from patients with and without ODE. The diagnosis was confirmed by neuro-ophthalmologists. Images with poor imaging qualities were excluded and after this exclusion, only the subjects with all four imaging modalities available on the record were included for the study. Any patients who had concurrent ODD-AION were included as NAION subjects based on the existence of ODE. Data was collected through following *en face* imaging modalities: (1) color fundus imaging; (2) FAF; (3) NIR reflectance imaging; (4) OCT (ONH and RNFL analysis).

#### 2.1.1 Multimodal imaging

We obtained the fundus color photographs and green-light FAF images using an Ultra-wide field Optos system (Optos PLC, Dunfermline, Scotland). This device uses confocal scanning laser ophthalmoscopy technology with a 200° field of view in a single capture. The green-light FAF used the ultrawide field Optomap-af function P200Tx with a field of view of up to 2008, optical resolution of 3,900 × 3,072 pixels, and acquisition time of 0.25 s/scan. According to the manufacturer’s design, the wide-field greenlight FAF and color fundus imaging of up to 2008 of retina are obtained in a single image. In addition, color fundus imaging was performed using Zeiss fundus camera model FF450 plus (Carl Zeiss Meditec, Jena, Germany). For FAF and NIR images, we used a confocal scanning laser ophthalmoscopy (cSLO) system (Spectralis HRA plus OCT, Heidelberg Engineering, Heidelberg, Germany). The blue-light FAF used a 308 × 308 field of view and an optical resolution of 1,536 × 1,536 pixels. According to the manufacturer’s design, the blue-light FAF intensity is adjustable by turning the sensitivity knob to optimize the image. NIR images were captured alone at 820 nm or simultaneously with OCT scans using a super luminescence diode to the central wavelength of 870 nm.

#### 2.1.2 OCT images acquisition and RNFL

Spectral-domain OCT (SD-OCT) images were acquired for the optic disc and macula using Cirrus HD-OCT (Carl Zeiss Meditec Inc., Jena, Germany) using a light source of 840 nm wavelength. The instrument has a maximum A-scan speed of 68,000 scans/s with an optical axial resolution of 5 mm and a scanning depth of 2 mm. We performed the Optic Disc Cube 200 × 200 acquiring 200 horizontal scan lines and Macular Cube 512 × 128 scans acquiring 128 horizontal scan lines. The thickness of the RNFL was automatically measured in a circle with a 3.46 mm diameter centered on the optic disc. The RNFL report demonstrated a summary of RNFL measurements, a thickness map, a deviation map, and multi-view tomograms of the disc area.

### 2.2 Training and grading

Two medical trainees (a senior medical student and a recent medical graduate) served as graders in this study. Prior to image grading, both underwent a structured 2-h training session led by a senior neuro-ophthalmologist. The training consisted of 20 example cases (5 each of NAION, ODD, anomalous optic discs, and healthy controls), presented with multimodal imaging including color fundus photography, FAF, NIR, and RNFL analysis. No clinical or demographic information was provided during training to preserve an image-only evaluation approach. Each modality was initially reviewed independently, followed by a slide presenting all modalities together for each case to reinforce comparative interpretation. The actual grading began 2 days after this training, and all training images were not part of the study’s prospectively collected data. For the main study, images were randomized by eye and grouped by modality. Each grader was separately presented with each image for a maximum of 15 s to grade the image on a scale of 0–5 (Table 1). The 15-s time limit was selected to encourage efficient, instinctive decision-making rather than prolonged deliberation, mimicking the time constraints commonly encountered in real-world clinical environments such as emergency or urgent care settings. To prevent bias, graders’ individual scores were masked between rounds, ensuring that scoring during subsequent modality reviews was independent. This was repeated for all individual modalities used, as well as various combinations of modalities (color fundus photography, FAF, NIR, RNFL, color fundus + FAF, color fundus + NIR, color fundus + RNFL, all modalities).

**Table 1:** Grading scale used by graders for each image.

Grading scale	
0	Definitely no edema
1	Likely no edema
2	Maybe no edema
3	Maybe edema
4	Likely edema
5	Definitely edema

### 2.3 Statistical design

#### 2.3.1 Accuracy of grades

The cases presented in this study can be accurately classified into one of two outcomes: (1) indicating true edema or (2) not indicating true edema. Images from healthy controls and patients with ODD, who exhibit no edema and pseudoedema respectively, should receive a grade of 0, while images from patients with NAION, who show true edema, should be graded as 5. Graders are allowed to assign scores ranging from 0 to 5, including 0 and 5. Weighted accuracy scores were then calculated based on the differences between the assigned score and the true grade. These weighted scores apply a mild penalty for uncertainty and a more significant penalty for incorrect outcomes. For instance, assigning a score of 3 to an actual grade of 5 results in a 70% accuracy (indicating a correct identification of ‘true edema’ but lacking confidence). In contrast, assigning a score of 2 to a true grade of 5 yields a 30% accuracy (indicating an incorrect classification of ‘no true edema,’ despite sharing the same confidence level as the score of 3).

Main effects of disease status, grader (between-subject factors) and imaging modality (within-subject factor) on grading accuracy 3 × 2 × 8 (disease status x grader x imaging modalities) were examined using a mixed ANOVA design. Main effects were considered significant at *p* ≤ 0.05. *Post-hoc* pairwise comparisons used Bonferroni correction (*α* = 0.05/number of comparisons) to control for multiple testing.

#### 2.3.2 Confidence of grades

Grading confidence was categorized based on the diagnostic certainty implied by each grade. Grades 0 and 5 were classified as high confidence, representing “definite” diagnoses of either no disc elevation (grade 0) or true ODE (grade 5). Grades 1 and 4 were categorized as medium confidence, indicating “likely” scenarios of no disc elevation or true ODE, respectively. Grades 2 and 3 were classified as low confidence, reflecting “maybe” assessments where graders were uncertain about the presence or absence of disc elevation.

#### 2.3.3 Chi-Square tests of independence

Chi-square tests of independence were performed to examine the relationship between confidence levels (high, medium, low) and: (1) true diagnosis (ODE, ODD, Control), (2) imaging modality, and (3) grader. Expected frequencies were verified to be >5 in all cells to ensure test validity. For significant chi-square results, standardized residuals were examined to identify cells contributing most to the association, with values >|2| considered noteworthy.

#### 2.3.4 Interrater reliability

In the current study, interrater reliability was calculated by assigning a score of ‘1’ when both raters provided identical ratings out of the six possible, indicating agreement. Conversely, a score of ‘0’ was assigned when the ratings between the two graders differed. The scores were then aggregated across cases within each disease status and image modality and the proportion of total cases that the two graders agreed on was calculated. Accuracy and confidence of the grades did not affect the interrater reliability scores.

## 3 Results

### 3.1 Study population

**Figure 1:**
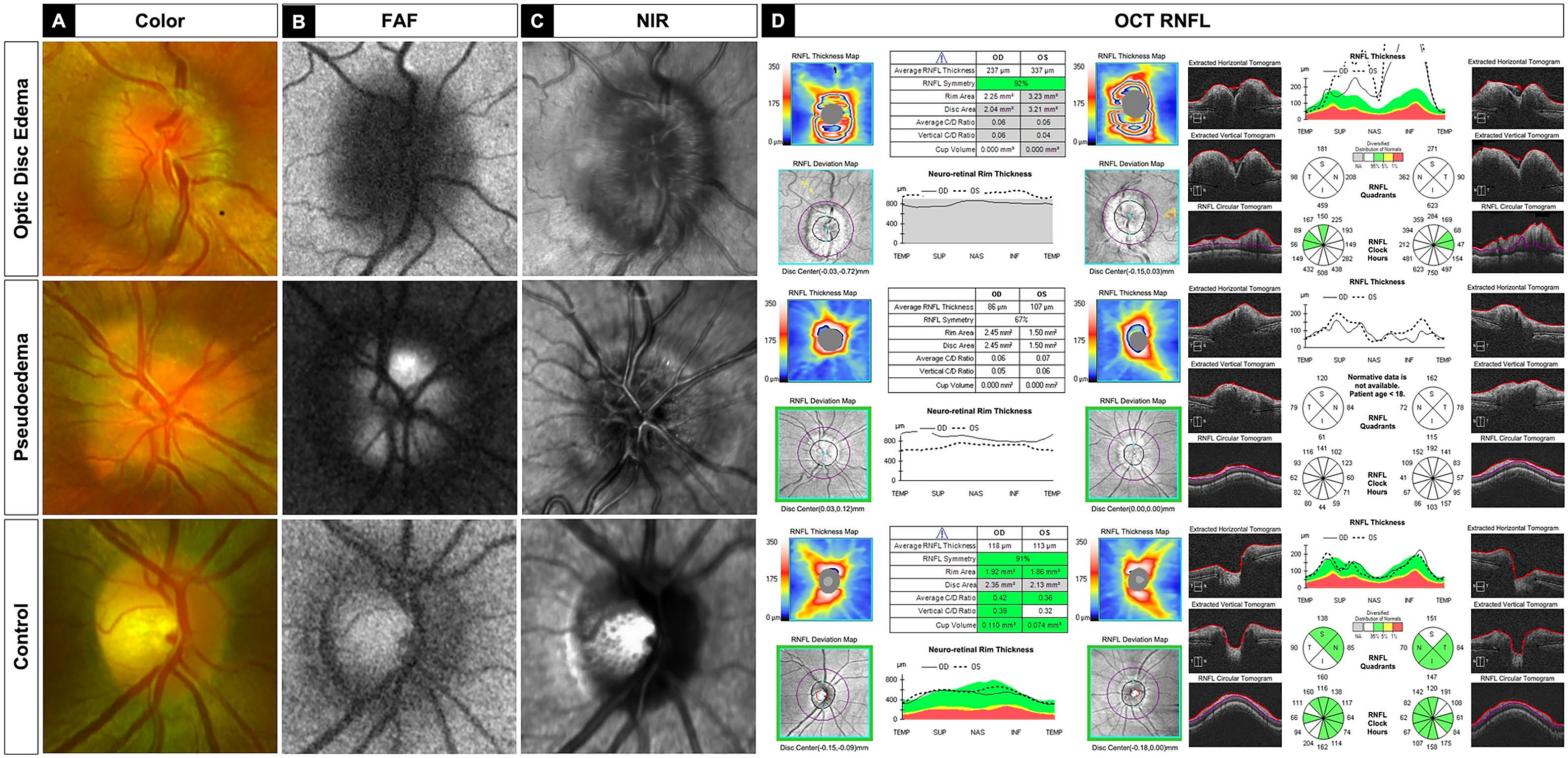
Representative examples of ODE (NAION), pseudoedema (ODD), and control images in **(A)** color fundus, **(B)** autofluorescence **(**FAF), **(C)** near-infrared reflectance (NIR), and **(D)** spectral-domain optical coherence tomography retinal nerve fiber layer (OCT-RNFL) analysis imaging modalities.

Images were collected from 98 subjects, encompassing a total of 149 eyes. Among these, there were 27 control subjects (30 eyes), 32 subjects with ODD (59 eyes), and 51 subjects with ODE/NAION (60 eyes). Table 2 shows subject demographics. The representative examples of ODE (NAION), ODD, and control images in each modality are shown in Figure 1.

**Table 2:** Study population demographics.

Study goup	Average age	Sex	Ethnicity (# of subjects)
Control	61.4 ± 16.65	M: 13 (43%) F: 17	White (15), Hispanic (6), Asian (5), Other (2), Declined/Unknown (2)
ODD	45.56 ± 21.91	M: 15 (25%) F: 44	White (37), Hispanic (7), Asian (2), Other (2), Unknown (11)
ODE/NAION	59.7 ± 15.39	M: 38 (63%) F: 22	White (37), Asian (4), Black (2), Other (10), Declined/Unknown (7)

### 3.2 Mixed ANOVA results for accuracy of grades

A 3 × 2 × 8 mixed ANOVA was conducted with Diagnosis (ODE, ODD, Control) and Grader as the between-subjects factor and Imaging Modality as a within-subjects factor.

#### 3.2.1 Main effects of between-subjects variables: diagnosis

There was a significant main effect of true diagnosis on grading accuracy: *F*(2, 160) = 6.076, *p* = 0.003. *Post-hoc* pairwise comparisons revealed that accuracy scores were significantly higher for control cases compared to both ODD (*t* = 4.48, *p* < 0.001) and ODE (*t* = 4.11, *p* < 0.001), while ODD and ODE were not significantly different (*t* = −0.71, *p* = 0.479). No significant main effect of Grader was observed: *F*(1, 160) = 0.177, *p* = 0.674, nor was there a significant Diagnosis × Grader interaction: *F*(2, 160) = 0.164, *p* = 0.849, indicating consistent ratings between the two graders across all diagnostic categories.

**Figure 2:**
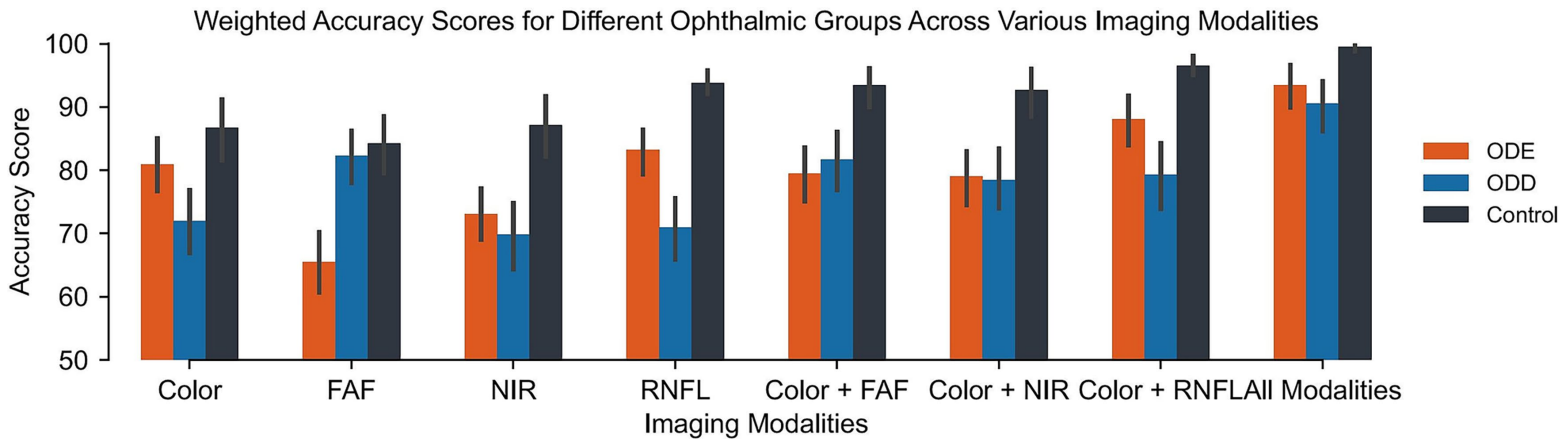
Weighted accuracy scores for ophthalmic groups when graded using various imaging modalities.

Grades assigned for control eyes were consistently the most accurate across all imaging modalities. For example, accuracy for controls was 86.7% with color imaging, 93.8% with RNFL, and reached 99.5% when all modalities were used. In contrast, ODD cases showed the lowest accuracy overall across most modalities, with 69.8% for NIR and 70.9% for RNFL, although FAF performed best among the single modalities at 82.3%. When all imaging modalities were used, ODD accuracy improved to 90.5%, showing a substantial benefit of combined imaging. ODE (NAION) cases fell in the middle, with accuracies ranging from 65.4% with FAF to 88.1% with color plus RNFL, and 93.4% using all modalities. However, the relationship is not proportionate: using color and another image only increases the accuracy slightly as compared to an image by itself. Weighted accuracy of grades comparison between different imaging modalities is shown in Figure 2 (descriptive statistics found in Supplementary Table S1). In general, multimodal imaging significantly improved the accuracy of diagnoses when compared to any of the single modalities or the combination of color and any of the other modalities (*p* < 0.001 with Bonferroni-correction, Table 3). Similarly, various combinations of color and other modalities, particularly color + RNFL, showed significant improvement in accuracy in comparison with single modalities. The full statistic results for all *post-hoc* pairwise comparisons (significant and non-significant) can be found in Supplementary Table S2.

**Table 3:** (A) Mixed-effects ANOVA results for diagnostic accuracy across imaging modalities. (B) *Post-hoc* pairwise comparisons of diagnostic accuracy between imaging modalities.

A					
Variables	SS	df	MS	*F*	*p*
Between-subject factors					
Intercept	1321642.14	1	1321642.14	7300.610	<0.001
Diagnosis	2199.99	2	1099.996	6.076	0.003
Grader	32.06	1	32.059	0.177	0.674
Diagnosis × grader	59.41	2	29.703	0.164	0.849
Error	28965.078	160	181.032		
Within-subject factors					
Modality	22186.971	4.867	4558.716	57.403	<0.001
Modality × diagnosis	6172.110	9.734	634.086	7.984	<0.001
Modality × grader	596.322	4.867	122.525	1.543	0.176
Modality *diagnosis* grader	416.369	9.734	42.775	0.539	0.859
Error (modality)	61841.856	778.71	79.416		
Analysis includes between-subjects factors [diagnosis: optic disc edema (ODE)/optic disc drusen (ODD)/control; grader: 1/2] and within-subjects factor (imaging modality: 8 conditions). Greenhouse–Geisser correction applied for violations of sphericity (*ε* = 0.695). SS, sum of squares; df, degrees of freedom; MS, mean square; *F*, *F*-statistic; *p*, probability value.					

B				
A	B	*t*	*p*	*p*-adj
All modalities	Color + FAF	6.59	<0.001	<0.001
All modalities	Color + NIR	8.30	<0.001	<0.001
All modalities	Color + RNFL	6.44	<0.001	<0.001
All modalities	FAF	8.15	<0.001	<0.001
All modalities	NIR	10.57	<0.001	<0.001
All modalities	RNFL	9.90	<0.001	<0.001
All modalities	Color	8.82	<0.001	<0.001
Color + RNFL	FAF	4.34	<0.001	0.001
Color + RNFL	NIR	7.08	<0.001	<0.001
Color + RNFL	RNFL	4.92	<0.001	<0.001
Color + RNFL	Color	6.56	<0.001	<0.001
Color + FAF	FAF	4.25	<0.001	0.001
Color + FAF	NIR	4.05	<0.001	0.002
Color + FAF	Color	3.67	<0.001	0.010
Color + NIR	NIR	4.11	<0.001	0.002
Color + NIR	Color + RNFL	−4.36	<0.001	0.001
NIR	RNFL	−3.53	0.001	0.016

Bonferroni-corrected paired *t*-tests following significant main effect of modality (*F*(4.867, 778.71) = 57.403, *p* < 0.001). Only comparisons with adjusted *p* < 0.050 shown (28 of 56 total comparisons). Upper section: All modalities versus other conditions; Middle section: Single versus dual-modality imaging; Lower section: Within category comparisons. Values represent *t*-statistics (df = 130) and adjusted *p*-values.

#### 3.2.2 Main effects of repeated-measures variables (imaging modality) and subsequent interactions

Mauchly’s Test indicated that the assumption of sphericity was violated (*W* = 0.159, *p* < 0.001). Therefore, values were adjusted using the Greenhouse–Geisser correction. Imaging modality had a significant effect on grading accuracy overall, *F*(4.67, 1152.26) = 8.29, *p* < 0.001, *ηp*^2^ = 0.032. Diagnoses performed using images from all modalities exhibited the highest mean accuracy at 94.27 (SE = 1.25), indicating strong performance. In contrast, the NIR-only modality yielded the lowest mean accuracy at 76.66 (SE = 1.61). Other combinations, such as color + FAF and color + RNFL, also demonstrated high mean accuracies of 88.03 and 85.18, respectively, with relatively low standard errors (SEs of 1.479 and 1.450), highlighting the variability in performance across different modalities.

**Figure 3:**
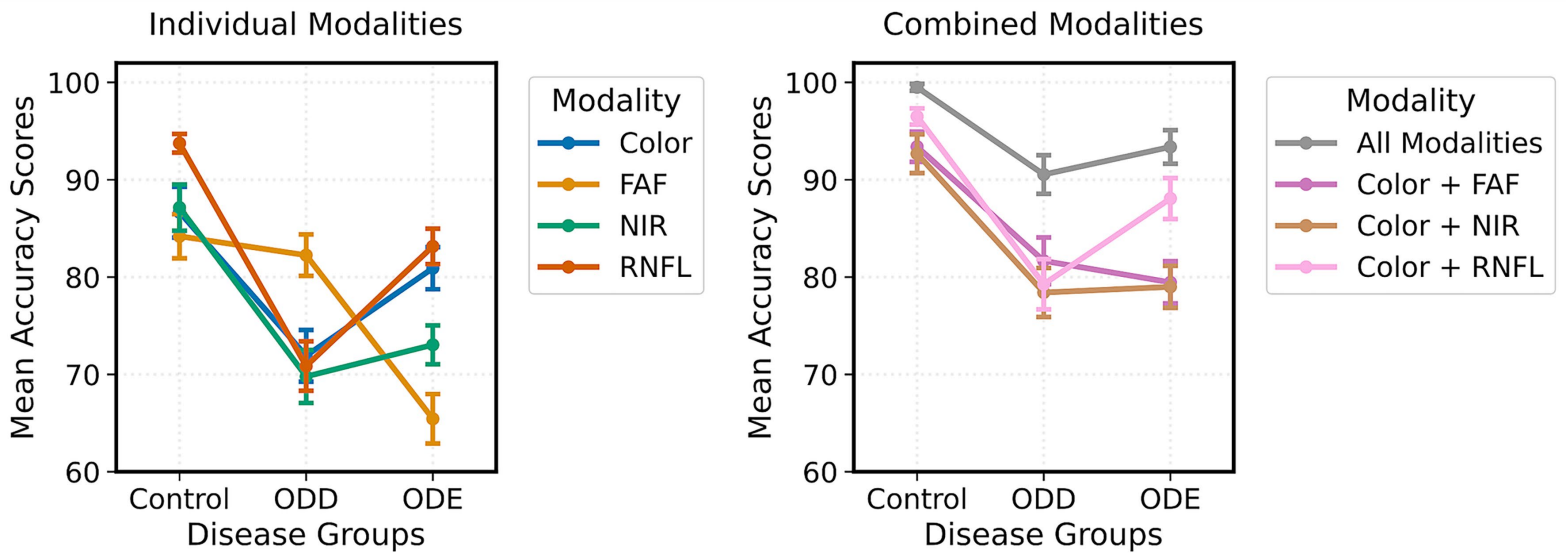
Interaction plot showing effect of disease group on mean accuracy scores for all imaging modalities. The plots mostly follow a similar trend, demonstrating a similar effect of disease groups on accuracy scores. The exception is FAF only and color + FAF, which lowered the accuracy scores when grading ODE images as compared to ODD.

There was a significant interaction between imaging modality and disease status, *F*(14, 896) = 6.746, *p* < 0.001, *ηp*^2^ = 0.095. For control subjects, all modalities and color + RNFL showed the greatest advantage over single modalities. For pathological cases (ODE and ODD), the benefit of multimodal imaging was even more pronounced, particularly when distinguishing ODD from ODE using FAF imaging (*t* = 3.85, *p* = 0.005) (Figure 3).

Multimodal imaging achieved the highest mean diagnostic accuracy (95.7%), followed by two-modality combinations (color + RNFL: 89.0%, color + NIR: 88.0%, color + FAF: 84.0%) and single modalities (RNFL: 86.3%, color: 83.7%, NIR: 83.3%, FAF: 80.0%). Notably, RNFL alone performed better than other single modalities. Control cases were diagnosed most accurately across all modalities, while ODD cases presented the greatest diagnostic challenge.

Bonferroni-corrected *post-hoc* tests revealed that diagnoses made using all four imaging modalities produced significantly higher accuracy than every other imaging modality combination (*p* < 0.001). Among two-modality combinations, color + RNFL showed significantly higher accuracy than all single modalities: FAF (*t* = 4.34, *p* < 0.001), NIR (*t* = 7.08, *p* < 0.001), and RNFL alone (*t* = 4.92, *p* < 0.001).

### 3.3 Confidence of grades

#### 3.3.1 Confidence patterns by diagnostic group and modality

**Figure 4:**
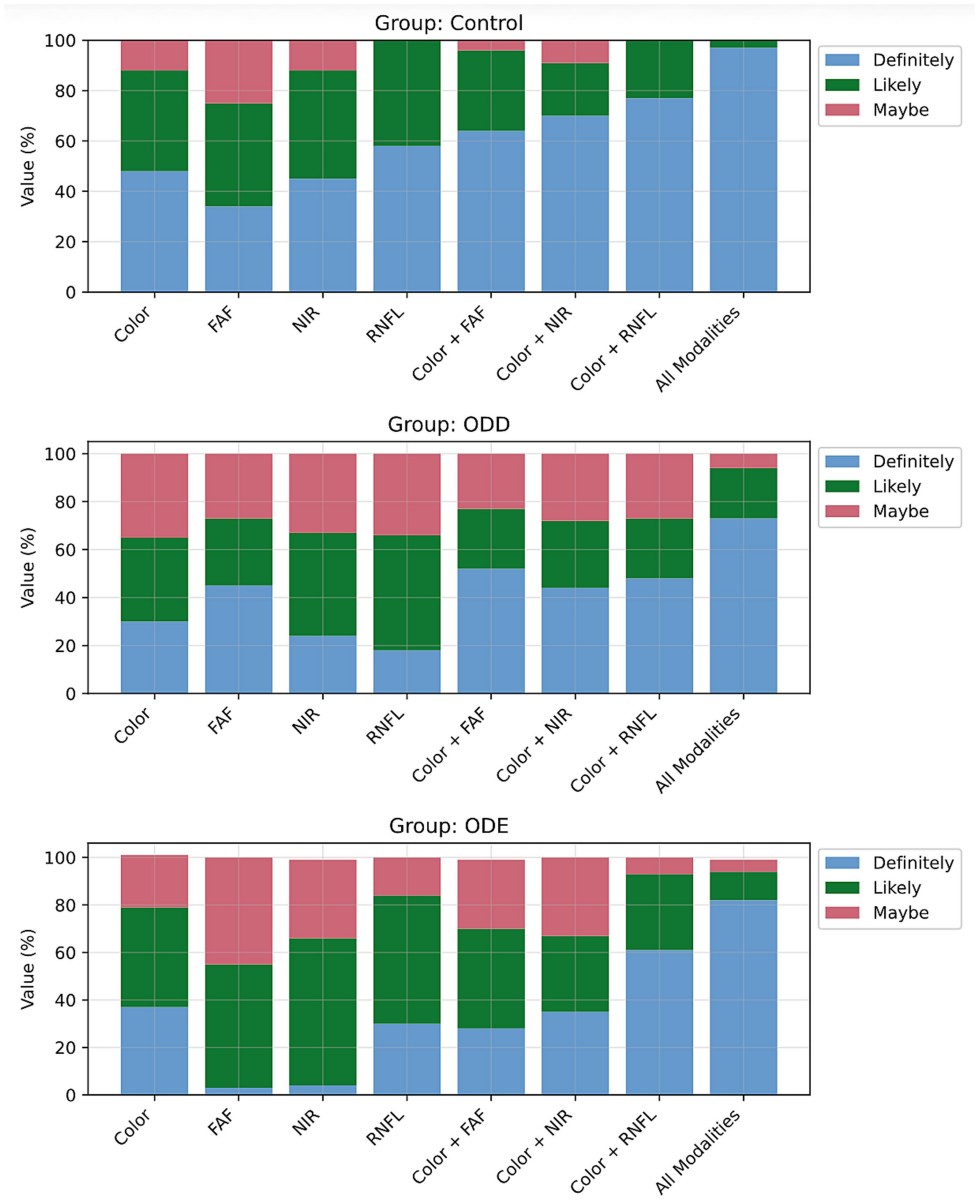
Confidence scores represented as a percentage of total cases (number of grades) assigned to each imaging modality.

Graders demonstrated consistently highest confidence when all modalities were used together across all diagnostic groups: 82% high confidence for ODE, 73% for ODD, and 97% for control cases (Figure 4, descriptive statistics found in Supplementary Table S3). Dual-modality combinations showed intermediate confidence levels, with color + RNFL achieving the greatest high confidence for ODE (61%) and control (77%) cases, while color + FAF performed best for ODD (52%). Single modality performance varied by diagnostic group: Color imaging yielded highest confidence for ODE (37%), FAF for ODD (45%), and RNFL for control cases (58%). However, single modalities generally resulted in substantial uncertainty, particularly for pathological cases. FAF alone showed notable limitations, producing the lowest high confidence percentage for controls (34%) and demonstrating significant uncertainty in ODE cases (52% medium confidence, 45% low confidence). Similarly, NIR imaging alone resulted in considerable uncertainty for both ODE (62% medium, 32% low confidence) and ODD (43% medium, 33% low confidence). RNFL alone yielded the lowest high confidence percentage for ODD cases (18%).

#### 3.3.2 Statistical validation of confidence patterns

Chi-square analysis provided strong statistical support for these clinical observations (Table 4). In comparing all modalities to single modalities, using all modalities together significantly improved confidence compared to individual modalities across all diagnostic groups (*p* < 0.001, Bonferroni corrected for all comparisons with Color, FAF, NIR, and RNFL). The full statistic chi-square independence results (significant and non-significant) can be found in Supplementary Table S4.

**Table 4:** Significant chi-square tests of independence for grader confidence.

Diagnosis	A	B	df	*Χ* ^2^	*p*
ODE	Color	FAF	2	40.214	<0.001
ODE	Color	NIR	2	39.011	<0.001
ODE	Color	Color + RNFL	2	17.596	<0.001
ODE	Color	All modalities	2	52.5	<0.001
ODE	FAF	RNFL	2	38.379	<0.001
ODE	FAF	Color + FAF	2	25.535	<0.001
ODE	FAF	Color + NIR	2	35.465	<0.001
ODE	FAF	Color + RNFL	2	91.096	<0.001
ODE	FAF	All modalities	2	141.83	<0.001
ODE	NIR	RNFL	2	31.009	<0.001
ODE	NIR	Color + FAF	2	25.758	<0.001
ODE	NIR	Color + NIR	2	41.243	<0.001
ODE	NIR	Color + RNFL	2	91.276	<0.001
ODE	NIR	All modalities	2	150.092	<0.001
ODE	RNFL	Color + RNFL	2	23.206	<0.001
ODE	RNFL	All modalities	2	66.345	<0.001
ODE	Color + FAF	Color + RNFL	2	29.762	<0.001
ODE	Color + FAF	All modalities	2	66.778	<0.001
ODE	Color + NIR	Color + RNFL	2	28.252	<0.001
ODE	Color + NIR	All modalities	2	58.154	<0.001
ODD	Color	All modalities	2	51.12	<0.001
ODD	FAF	RNFL	2	20.526	<0.001
ODD	FAF	All modalities	2	25.539	<0.001
ODD	NIR	Color + FAF	2	19.214	<0.001
ODD	NIR	Color + RNFL	2	15.505	<0.001
ODD	NIR	All modalities	2	60.905	<0.001
ODD	RNFL	Color + FAF	2	29.86	<0.001
ODD	RNFL	Color + NIR	2	19.653	<0.001
ODD	RNFL	Color + RNFL	2	25.4	<0.001
ODD	RNFL	All modalities	2	75.262	<0.001
ODD	Color + FAF	All modalities	2	17.561	<0.001
ODD	Color + NIR	All modalities	2	26.961	<0.001
ODD	Color + RNFL	All modalities	2	22.504	<0.001
Control	Color	All modalities	2	35.282	<0.001
Control	FAF	RNFL	2	18.708	<0.001
Control	FAF	Color + FAF	2	14.864	0.001
Control	FAF	Color + RNFL	2	27.299	<0.001
Control	FAF	All modalities	2	51.316	<0.001
Control	NIR	Color + RNFL	2	15.724	<0.001
Control	NIR	All modalities	2	38.644	<0.001
Control	RNFL	All modalities	1	23.13	<0.001
Control	Color + FAF	All modalities	2	19.835	<0.001
Control	Color + NIR	All modalities	2	15.745	<0.001

Association between imaging modality and grader confidence levels within each diagnostic category. Chi-square tests of independence revealed relationships between imaging modality and confidence ratings (definitely, likely, maybe) across all pairwise comparisons. Results are stratified by true diagnosis [optic disc edema (ODE), optic disc drusen (ODD), control]. Table only includes significant comparisons. For all comparisons, see Supplementary Table S4. df, degrees of freedom; *X*^2^, chi-square statistic; *p*, probability value.

Diagnostic group differences emerged in the statistical patterns. ODE cases showed the most pronounced statistical differences between imaging modalities, with numerous significant comparisons surviving correction for multiple testing, indicating that modality choice has particularly strong impact on confidence when evaluating optic disc edema. ODD cases demonstrated moderate but consistent patterns of statistical significance. In contrast, control subjects showed fewer significant differences between modalities, aligning with their relative diagnostic clarity regardless of imaging method.

Single modality comparisons revealed significant differences supporting clinical observations. FAF vs. RNFL comparisons showed strong statistical significance in both ODE and ODD groups (*p* < 0.001), validating the finding that FAF achieved higher confidence in ODD cases (45%) while RNFL was superior for controls (58%). In dual-modality validation, statistical comparisons between combined modalities (color + FAF, color + NIR, color + RNFL) and single modalities frequently showed significant differences, particularly for pathological cases (*p* < 0.001). These results support the clinical finding that color + RNFL achieved highest confidence among two-modality methods for both ODE (61%) and controls (77%).

The statistical evidence strongly supports that comprehensive multi-modal imaging protocols provide significantly higher diagnostic confidence, with the effect being most pronounced in pathological cases where careful differentiation is crucial.

### 3.4 Interrater reliability

The highest interrater reliability for the control group was achieved with the combination of all modalities (100%), followed by RNFL alone (90.0%) and the combination of color and RNFL (86.7%). For the ODD group, the highest reliability was observed with the combination of all modalities (88.1%), with the next best being color and NIR (78.0%). For the ODE group, the highest reliability was also with all modalities combined (91.7%), followed by color and RNFL (85.0%). The results (Supplementary Table S5) indicate that combining multiple imaging modalities generally improves interrater reliability across all diagnostic categories, with the combination of all modalities yielding the highest reliability scores.

## 4 Discussion

Accurate diagnosis of ODE is an important part of neuro-ophthalmic assessment and a diagnostic challenge. We performed a prospective case-control study of 149 eyes in 98 subjects to investigate the accuracy and confidence of diagnosing NAION, ODD, or healthy controls using single vs. multimodal imaging. Overall, diagnostic accuracy using single modality, especially color and RNFL, was adequate for ONHE detection. However, using multimodal imaging substantially increased both diagnostic accuracy and confidence. Detecting ONHE with true ODE, such as in NAION, papilledema, and optic neuritis (19, 20), from pseudoedema like ODD and congenital optic disc anomalies, is crucial for urgent and comprehensive evaluation.

Color fundus images alone had better diagnostic accuracy (80.9%) for NAION compared to other single modalities such as FAF (65.4%) and NIR (73.0%) images, demonstrating that ODE from NAION can be validly confirmed based on color fundus images, just like the use of the Frisen scale in papilledema (21). Diagnostic accuracy of ODE using color fundus photography relies on specific distortions of the optic disc, which can be further broken down using diagnostic descriptors, such as swelling from thickening of the peripapillary RNFL (2) and halo sign (22). In a separate effort, the Brain and Optic Nerve Study with Artificial Intelligence (BONSAI) group also showed high diagnostic accuracy for papilledema (87.5%), non-papilledema optic disc abnormalities (81.1%), and controls (91.8%) using color fundus imaging alone (23). While artificial intelligence (AI)-based interpretation of single images holds promise, our study highlights how combining imaging modalities can enhance human interpretation, particularly in diagnostically ambiguous cases where AI tools may not yet be validated or available.

Similar to color fundus imaging, the OCT RNFL analysis alone had a high diagnostic accuracy of 83.2% for the presence of ODE. The most common OCT machines are equipped with specialized protocols to analyze the optic nerve head. For example, the OCT ONH and RNFL analysis uses a circular scan in the peripapillary region to provide qualitative heatmap of the optic nerve head, automatic segmentation and quantitative measurements of the optic disc features and peripapillary RNFL thickness (24). Review of such OCT reports should start with evaluation of the signal strength and position of the segmentation lines, which are included in the report. Once there is visual confirmation of the accuracy of the measurements, the mean RNFL thickness may be enough to diagnose and monitor ODE over time (25).

Qualitative OCT B-scan through the optic nerve head without RNFL analysis may also be sufficient to distinguish cases of pseudoedema such as ODD vs. ODE with 63% sensitivity (26). Johnson et al. (26) describe a recumbent V-shaped hyporeflective space next to the optic disc on OCT to distinguish ODE and ODD. ODE is associated with the presence of subretinal spaces between the sensory retina and the retinal pigment epithelium and choriocapillaris complex pattern, while ODD has an abrupt decline of the hyporeflective space. RNFL thickness was also found to be significantly greater in ODE (176.3–247.2 μm) compared to ODD (78.6–153.8 μm) in all four quadrants. Carta et al. (27) found that using cutoff values of ≥ 110 μm from the average of all four quadrants or ≥ 150 μm from the inferior quadrant RNFL thickness can diagnose ODE from pseudoedema with 70% accuracy. However, the auto segmentation algorithm can be poor in ODE, so any interpretation of the RNFL analysis can only be done after confirming high signal strength and segment of the RNFL layer.

Our study shows accuracy of 73.0% for NIR alone and 65.4% for FAF alone for NAION. Although NIR and FAF are essentially never interpreted alone as a single modality, they are often combined with color fundus imaging. NIR can distinguish ODD based on their clustered, hyper-reflectance of optic disc margins (28, 29). Combined with color fundus imaging, FAF helps identify contributors to ONHE due to optic disc drusen. Our previous study found green-light FAF to have the highest sensitivity in ODD diagnosis compared to blue-light, NIR, and color (30). This further supports previous findings of green-light FAF imaging having high sensitivity (87.8%) and specificity (100%) for detecting superficial ODD (31). However, due to FAF’s low image resolution and inability to detect hyper autofluorescence from buried drusen, the enhanced depth imaging OCT (EDI-OCT) remains the gold standard in true diagnosis of ODD (32, 33). Nevertheless, FAF use was correlated with high confidence for 45% of single FAF modality ODD diagnosis and 52% with color + FAF in ODD diagnosis by the graders, which was the highest confidence score for ODD in single- and dual-modality groups. Hence, in settings that require emergent interpretation, FAF is able to provide relevant ODD information for non-ophthalmologists.

As NAION is the most common cause of vision loss for patients with ODD (34), FAF-confirmed ODD alone cannot rule out superimposed ODD-AION. Accordingly, the high diagnostic confidence observed with single-modality FAF (45%) and two-modality color + FAF (52%) for ODD was approached with caution until all imaging modalities were used together, at which point confidence increased to 73%. The presence of ODD is especially worrisome due to a crowded disc-at-risk configuration that places patients at higher risk for ischemic events and vision loss (12, 14). Although no FDA-approved treatment currently exists for either NAION or ODD, early detection is critical: up to 15% of patients with NAION will experience vision loss in the contralateral eye within 5 years (7). Therefore, identifying at-risk individuals enables clinicians to implement close monitoring and control of modifiable risk factors such as obstructive sleep apnea and systemic vascular conditions (35). As such, using multimodal imaging for simultaneous assessment for both structural signs of ODD and active optic disc edema can address the limitations of individual imaging modalities to provide more holistic, accurate clinical decision-making.

High diagnostic accuracy using multimodal imaging was accompanied by high confidence scores across all diagnostic categories, including control (97%), NAION (82%), and ODD (73%). Importantly, the increased confidence in diagnosing true ODE was accompanied by accuracy. In only one case of true ODE, the two graders had higher confidence in falsely ruling out ODE. While diagnostic accuracy is often reported, fewer studies explore diagnostic confidence, which plays a critical role in clinical decision-making, particularly when evaluating ONHE in urgent settings. Many patients with ONHE due to ODE initially present to emergency departments (EDs) or general clinics, where ophthalmic expertise is limited. Prior studies have shown that emergency physicians and non-ophthalmology trainees often lack confidence in evaluating fundus findings due to limited training in ophthalmoscopy and image interpretation (36, 37). In our study, the two medical trainees’ performance reflects the diagnostic potential of multimodal imaging in the hands of general physicians and non-ophthalmology specialists, who are often the first point of contact for patients with visual complaints. Importantly, multimodal imaging significantly improved diagnostic accuracy compared to single or dual modalities, reaching 99.5% for controls, 93.4% for NAION, and 90.5% for ODD, suggesting that such tools could enhance early triage and referral accuracy even among providers without subspecialty training.

Although not directly assessed in this study, multimodal imaging may hold future value in distinguishing pseudopapilledema from true papilledema—an area where diagnostic uncertainty often leads to unnecessary testing. This could be particularly useful in pediatric patients with idiopathic intracranial hypertension (IIH), among whom the incidence of ODD is 15–48% (38, 39), compared to approximately 2% in the general population (8, 9). In such cases, the presence of ODD may obscure or complicate the diagnosis of papilledema, and its association with crowded optic nerves may increase susceptibility to ischemic damage. Prior studies have found that high-grade papilledema, rather than ODD itself, is more strongly associated with permanent vision loss (38). These observations underscore the clinical value of future investigations into how multimodal imaging might enhance diagnostic accuracy and reduce morbidity in patients with overlapping or ambiguous optic disc findings.

There are several limitations in the study. First, NAION and ODD were used as representative models for true ODE and pseudoedema, respectively. We focused on acute NAION because it is the most common cause of acute optic neuropathy in patients older than 50 years of age and the leading cause of vision loss in patients with ODD (3, 5). However, the results cannot be reliably applied to other important causes of optic disc edema without further studies. Assessing the use of multimodal imaging in other causes, such as papilledema and optic neuritis, can be particularly beneficial as they frequently drive urgent consultation.

Second, the demographic profile of our cohort reflects an imbalance across several dimensions. Most subjects were White, limiting the generalizability of our results to more diverse populations. Likewise, the sex distribution differed between disease groups: 63% of NAION subjects were male, and 75% of ODD subjects were female. While this pattern aligns with known epidemiologic trends in these conditions (40, 41), it raises the possibility of sex-linked structural differences in the optic nerve that could influence image-based diagnosis. However, given that the graders were fully blinded to all patient demographics, any potential sex-related anatomical variations could not have biased grader decisions through prior knowledge or expectation. Therefore, the biological differences in optic nerve morphology between sexes were not statistically adjusted in the analysis.

Lastly, our study does not investigate multimodal imaging in children and includes very few young adults, who may present with greater diagnostic difficulties due to ODD being more likely to be buried and less calcified to be detected with FAF (42, 43). It will be important for future studies to focus on subjects less than 50 years old, since ODD is the most common pseudoedema associated with younger onset NAION without other vascular risk factors (13). Additional considerations for future work include assessing intra-grader reliability and evaluating how time elapsed since training affects diagnostic performance.

In summary, our study supports the role of multimodal imaging in evaluating the optic nerve in urgent care settings, where non-neuro-ophthalmologists often serve as first-line responders for ODE-related emergencies. Prior ED studies using non-mydriatic fundus photography combined with OCT have shown that multimodal imaging improves diagnostic accuracy, facilitates timely triage, and reduces unnecessary testing and provider workload, ultimately benefiting patient outcomes and lowering healthcare costs (44–46). These findings also highlight the potential utility of multimodal imaging in teleophthalmology and remote care environments, where image quality and examiner expertise may be variable. By enabling more confident and accurate assessments across a range of care settings, multimodal imaging offers a scalable strategy to improve decision-making for patients presenting with optic nerve head elevation. Future research should further investigate factors that influence not only diagnostic accuracy but also clinician confidence when evaluating abnormal optic discs.

## Data Availability

The raw data supporting the conclusions of this article will be made available by the authors, without undue reservation.
