# Supplementary material for: Using multimodal imaging to improve the diagnostic accuracy and confidence in distinguishing non-arteritic anterior ischemic optic neuropathy from optic disc drusen
Source: Front Neurol. 2026 Feb 10;17:1653402. doi: 10.3389/fneur.2026.1653402 (PMC12929115; doi:10.3389/fneur.2026.1653402)
Supplement: Supplementary file 1 [file Table_1.DOCX]

Supplementary Material

# Supplementary Tables

**Supplementary Table S1.** Descriptive statistics for weighted accuracy scores by imaging modality and diagnosis.

|  | ODE | ODD | Control |
| --- | --- | --- | --- |
| Sample Size | N = 51  (60 cases) | N = 32  (59 cases) | N = 27  (30 cases) |
| Color | 80.9 (23.72) | 71.9 (29.08) | 86.7 (20.18) |
| FAF | 65.4 (25.66) | 82.3 (23.31) | 84.2 (16.94) |
| NIR | 73.0 (21.82) | 69.8 (29.68) | 87.1 (17.81) |
| RNFL | 83.2 (19.46) | 70.9 (27.38) | 93.8 (7.46) |
| Color + FAF | 79.5 (21.95) | 81.7 (26.35) | 93.4 (11.6) |
| Color + NIR | 79.0 (23.66) | 78.4 (27.57) | 92.7 (14.89) |
| Color + RNFL | 88.1 (22.47) | 79.3 (27.75) | 96.5 (6.4) |
| All Modalities | 93.4 (18.9) | 90.5 (21.73) | 99.5 (2.72) |
| *Mean (SD) accuracy scores for N = 149 eyes (Optic Disc Edema [ODE]: n = 51, Optic Disc Drusen [ODD]: n = 49, Control: n = 49) across eight imaging modality conditions. Scores range from 0-100, with higher values indicating greater diagnostic accuracy. Each case rated by 2 independent graders.* | | | |

**Supplementary Table S2.** Complete post-hoc pairwise comparisons of diagnostic accuracy between all imaging modality combinations.

| Contrast | Modality | A | B | T | df | p | p-corr |
| --- | --- | --- | --- | --- | --- | --- | --- |
| Diagnosis | - | ODD | ODE | -0.79 | 112 | 112 | 0.430 |
| Diagnosis | - | Control | ODE | 4.08 | 87 | 87 | 0.000 |
| Diagnosis | - | Control | ODD | 4.43 | 82 | 82 | 0.000 |
| Modality | - | NIR | RNFL | -4.08 | 141 | 141 | 0.000 |
| Modality | - | FAF | RNFL | -1.64 | 132 | 132 | 0.103 |
| Modality | - | FAF | NIR | 0.77 | 135 | 135 | 0.442 |
| Modality | - | Color | RNFL | -1.28 | 143 | 143 | 0.204 |
| Modality | - | Color | NIR | 2.12 | 146 | 146 | 0.035 |
| Modality | - | Color | FAF | 0.78 | 137 | 137 | 0.435 |
| Modality | - | Color | Color+RNFL | -6.54 | 143 | 143 | 0.000 |
| Modality | - | Color | Color+NIR | -2.69 | 146 | 146 | 0.008 |
| Modality | - | Color | Color+FAF | -3.80 | 137 | 137 | 0.000 |
| Modality | - | Color+RNFL | RNFL | 5.09 | 143 | 143 | 0.000 |
| Modality | - | Color+RNFL | NIR | 7.39 | 141 | 141 | 0.000 |
| Modality | - | Color+RNFL | FAF | 4.45 | 132 | 132 | 0.000 |
| Modality | - | Color+NIR | RNFL | 0.57 | 141 | 141 | 0.569 |
| Modality | - | Color+NIR | NIR | 4.67 | 146 | 146 | 0.000 |
| Modality | - | Color+NIR | FAF | 2.25 | 135 | 135 | 0.026 |
| Modality | - | Color+FAF | RNFL | 1.33 | 132 | 132 | 0.184 |
| Modality | - | Color+FAF | NIR | 4.09 | 135 | 135 | 0.000 |
| Modality | - | Color+FAF | FAF | 4.46 | 137 | 137 | 0.000 |
| Modality | - | Color+NIR | Color+RNFL | -4.23 | 141 | 141 | 0.000 |
| Modality | - | Color+FAF | Color+RNFL | -2.35 | 132 | 132 | 0.020 |
| Modality | - | Color+FAF | Color+NIR | 1.89 | 135 | 135 | 0.061 |
| Modality | - | All Modalities | RNFL | 10.22 | 143 | 143 | 0.000 |
| Modality | - | All Modalities | NIR | 11.16 | 146 | 146 | 0.000 |
| Modality | - | All Modalities | FAF | 8.40 | 137 | 137 | 0.000 |
| Modality | - | All Modalities | Color | 9.22 | 148 | 148 | 0.000 |
| Modality | - | All Modalities | Color+RNFL | 6.58 | 143 | 143 | 0.000 |
| Modality | - | All Modalities | Color+NIR | 8.55 | 146 | 146 | 0.000 |
| Modality | - | All Modalities | Color+FAF | 6.73 | 137 | 137 | 0.000 |
| Modality * Diagnosis | All Modalities | ODD | ODE | -0.80 | 114 | 114 | 0.423 |
| Modality * Diagnosis | All Modalities | Control | ODE | 2.46 | 63 | 63 | 0.016 |
| Modality * Diagnosis | All Modalities | Control | ODD | 3.18 | 61 | 61 | 0.002 |
| Modality * Diagnosis | Color | ODD | ODE | -2.02 | 110 | 110 | 0.046 |
| Modality * Diagnosis | Color | Control | ODE | 1.40 | 78 | 78 | 0.166 |
| Modality * Diagnosis | Color | Control | ODD | 3.26 | 85 | 85 | 0.002 |
| Modality * Diagnosis | Color+FAF | ODD | ODE | 0.43 | 107 | 107 | 0.670 |
| Modality * Diagnosis | Color+FAF | Control | ODE | 4.02 | 75 | 75 | 0.000 |
| Modality * Diagnosis | Color+FAF | Control | ODD | 3.17 | 82 | 82 | 0.002 |
| Modality * Diagnosis | Color+NIR | ODD | ODE | -0.21 | 112 | 112 | 0.835 |
| Modality * Diagnosis | Color+NIR | Control | ODE | 3.53 | 79 | 79 | 0.001 |
| Modality * Diagnosis | Color+NIR | Control | ODD | 3.34 | 84 | 84 | 0.001 |
| Modality * Diagnosis | Color+RNFL | ODD | ODE | -1.98 | 112 | 112 | 0.051 |
| Modality * Diagnosis | Color+RNFL | Control | ODE | 2.70 | 69 | 69 | 0.009 |
| Modality * Diagnosis | Color+RNFL | Control | ODD | 4.70 | 65 | 65 | 0.000 |
| Modality * Diagnosis | FAF | ODD | ODE | 3.90 | 101 | 101 | 0.000 |
| Modality * Diagnosis | FAF | Control | ODE | 4.26 | 74 | 74 | 0.000 |
| Modality * Diagnosis | FAF | Control | ODD | 0.50 | 70 | 70 | 0.620 |
| Modality * Diagnosis | NIR | ODD | ODE | -0.90 | 105 | 105 | 0.373 |
| Modality * Diagnosis | NIR | Control | ODE | 3.38 | 63 | 63 | 0.001 |
| Modality * Diagnosis | NIR | Control | ODD | 3.71 | 80 | 80 | 0.000 |
| Modality * Diagnosis | RNFL | ODD | ODE | -3.00 | 112 | 112 | 0.003 |
| Modality * Diagnosis | RNFL | Control | ODE | 3.73 | 78 | 78 | 0.000 |
| Modality * Diagnosis | RNFL | Control | ODD | 6.34 | 70 | 70 | 0.000 |

*Full results of Bonferroni-corrected paired t-tests (56 comparisons, adjusted α = .05/28 = .0018) comparing weighted accuracy scores between all pairs of imaging modalities. Shaded rows indicate statistically significant differences (adjusted p < .050). Positive t-values indicate higher accuracy for Modality A versus Modality B. T = T-statistic; df = degree of freedom; p = uncorrected p-value; p-corr = corrected p-value.*

**Supplementary Table S3.** Distribution of confidence levels across imaging modalities and diagnostic categories

| Imaging Modality | *ODE* | | | | | *ODD* | | | | | *Control* | | | | |  |
| --- | --- | --- | --- | --- | --- | --- | --- | --- | --- | --- | --- | --- | --- | --- | --- | --- |
|  | Definitely | Likely | | Maybe | | Definitely | Likely | | Maybe | | Definitely | Likely | | Maybe | |  |
| Color | 44 (37%) | | 50 (42%) | | 26 (22%) | 36 (30%) | | 42 (35%) | | 42 (35%) | 29 (48%) | | 24 (40%) | | 7  (12%) | |
| FAF | 3  (3%) | | 53 (52%) | | 46 (45%) | 54 (45%) | | 34 (28%) | | 32 (27%) | 19 (34%) | | 23 (41%) | | 14  (25%) | |
| NIR | 5  (4%) | | 75 (62%) | | 40 (33%) | 29 (24%) | | 51 (43%) | | 39 (33%) | 25 (45%) | | 24 (43%) | | 7  (12%) | |
| RNFL | 34 (30%) | | 62 (54%) | | 18 (16%) | 21 (18%) | | 56 (48%) | | 39 (34%) | 35 (58%) | | 25 (42%) | | 0  (0%) | |
| Color + FAF | 29 (28%) | | 43 (42%) | | 30 (29%) | 62 (52%) | | 30 (25%) | | 28 (23%) | 36 (64%) | | 18 (32%) | | 2  (4%) | |
| Color + NIR | 42 (35%) | | 38 (32%) | | 40 (33%) | 53 (44%) | | 34 (28%) | | 33 (28%) | 39 (70%) | | 12 (21%) | | 5  (9%) | |
| Color + RNFL | 70 (61%) | | 36 (32%) | | 8  (7%) | 56 (48%) | | 29 (25%) | | 31 (27%) | 46 (77%) | | 14 (23%) | | 0  (0%) | |
| All  Modalities | 99 (82%) | | 15 (12%) | | 6  (5%) | 88 (73%) | | 25 (21%) | | 7  (6%) | 58 (97%) | | 2  (3%) | | 0  (0%) | |

*Frequency counts and percentages of grader confidence ratings (High: grades 0,5; Medium: grades 1,4; Low: grades 2,3) for each combination of true diagnosis (Optic Disc Edema [ODE], Optic Disc Drusen [ODD], Control) and imaging modality. Data represent 298 grading instances (149 eyes × 2 graders) per modality-diagnosis combination. Percentages calculated within each modality-diagnosis cell.*

**Supplementary Table S4.** Complete chi-square analyses of confidence level distributions between all imaging modality pairs by diagnosis.

| Diagnosis | A | B | df | Χ^2^ | p |
| --- | --- | --- | --- | --- | --- |
| ODE | Color | FAF | 2 | 40.214 | 0 |
| ODE | Color | NIR | 2 | 39.011 | 0 |
| ODE | Color | RNFL | 2 | 3.871 | 0.144 |
| ODE | Color | Color + FAF | 2 | 2.451 | 0.294 |
| ODE | Color | Color + NIR | 2 | 4.653 | 0.098 |
| ODE | Color | Color + RNFL | 2 | 17.596 | 0 |
| ODE | Color | All Modalities | 2 | 52.5 | 0 |
| ODE | FAF | NIR | 2 | 3.262 | 0.196 |
| ODE | FAF | RNFL | 2 | 38.379 | 0 |
| ODE | FAF | Color + FAF | 2 | 25.535 | 0 |
| ODE | FAF | Color + NIR | 2 | 35.465 | 0 |
| ODE | FAF | Color + RNFL | 2 | 91.096 | 0 |
| ODE | FAF | All Modalities | 2 | 141.83 | 0 |
| ODE | NIR | RNFL | 2 | 31.009 | 0 |
| ODE | NIR | Color + FAF | 2 | 25.758 | 0 |
| ODE | NIR | Color + NIR | 2 | 41.243 | 0 |
| ODE | NIR | Color + RNFL | 2 | 91.276 | 0 |
| ODE | NIR | All Modalities | 2 | 150.092 | 0 |
| ODE | RNFL | Color + FAF | 2 | 6.187 | 0.045 |
| ODE | RNFL | Color + NIR | 2 | 14.803 | 0.001 |
| ODE | RNFL | Color + RNFL | 2 | 23.206 | 0 |
| ODE | RNFL | All Modalities | 2 | 66.345 | 0 |
| ODE | Color + FAF | Color + NIR | 2 | 2.676 | 0.262 |
| ODE | Color + FAF | Color + RNFL | 2 | 29.762 | 0 |
| ODE | Color + FAF | All Modalities | 2 | 66.778 | 0 |
| ODE | Color + NIR | Color + RNFL | 2 | 28.252 | 0 |
| ODE | Color + NIR | All Modalities | 2 | 58.154 | 0 |
| ODE | Color + RNFL | All Modalities | 2 | 13.764 | 0.001 |
| ODD | Color | FAF | 2 | 5.793 | 0.055 |
| ODD | Color | NIR | 2 | 1.732 | 0.421 |
| ODD | Color | RNFL | 2 | 5.992 | 0.05 |
| ODD | Color | Color + FAF | 2 | 11.698 | 0.003 |
| ODD | Color | Color + NIR | 2 | 5.169 | 0.075 |
| ODD | Color | Color + RNFL | 2 | 8.32 | 0.016 |
| ODD | Color | All Modalities | 2 | 51.12 | 0 |
| ODD | FAF | NIR | 2 | 11.616 | 0.003 |
| ODD | FAF | RNFL | 2 | 20.526 | 0 |
| ODD | FAF | Color + FAF | 2 | 1.068 | 0.586 |
| ODD | FAF | Color + NIR | 2 | 0.025 | 0.988 |
| ODD | FAF | Color + RNFL | 2 | 0.381 | 0.826 |
| ODD | FAF | All Modalities | 2 | 25.539 | 0 |
| ODD | NIR | RNFL | 2 | 1.476 | 0.478 |
| ODD | NIR | Color + FAF | 2 | 19.214 | 0 |
| ODD | NIR | Color + NIR | 2 | 10.92 | 0.004 |
| ODD | NIR | Color + RNFL | 2 | 15.505 | 0 |
| ODD | NIR | All Modalities | 2 | 60.905 | 0 |
| ODD | RNFL | Color + FAF | 2 | 29.86 | 0 |
| ODD | RNFL | Color + NIR | 2 | 19.653 | 0 |
| ODD | RNFL | Color + RNFL | 2 | 25.4 | 0 |
| ODD | RNFL | All Modalities | 2 | 75.262 | 0 |
| ODD | Color + FAF | Color + NIR | 2 | 1.364 | 0.506 |
| ODD | Color + FAF | Color + RNFL | 2 | 0.407 | 0.816 |
| ODD | Color + FAF | All Modalities | 2 | 17.561 | 0 |
| ODD | Color + NIR | Color + RNFL | 2 | 0.474 | 0.789 |
| ODD | Color + NIR | All Modalities | 2 | 26.961 | 0 |
| ODD | Color + RNFL | All Modalities | 2 | 22.504 | 0 |
| Control | Color | FAF | 2 | 4.305 | 0.116 |
| Control | Color | NIR | 2 | 0.159 | 0.924 |
| Control | Color | RNFL | 2 | 7.583 | 0.023 |
| Control | Color | Color + FAF | 2 | 4.256 | 0.119 |
| Control | Color | Color + NIR | 2 | 5.673 | 0.059 |
| Control | Color | Color + RNFL | 2 | 13.485 | 0.001 |
| Control | Color | All Modalities | 2 | 35.282 | 0 |
| Control | FAF | NIR | 2 | 3.173 | 0.205 |
| Control | FAF | RNFL | 2 | 18.708 | 0 |
| Control | FAF | Color + FAF | 2 | 14.864 | 0.001 |
| Control | FAF | Color + NIR | 2 | 14.617 | 0.001 |
| Control | FAF | Color + RNFL | 2 | 27.299 | 0 |
| Control | FAF | All Modalities | 2 | 51.316 | 0 |
| Control | NIR | RNFL | 2 | 8.559 | 0.014 |
| Control | NIR | Color + FAF | 2 | 5.619 | 0.06 |
| Control | NIR | Color + NIR | 2 | 7.396 | 0.025 |
| Control | NIR | Color + RNFL | 2 | 15.724 | 0 |
| Control | NIR | All Modalities | 2 | 38.644 | 0 |
| Control | RNFL | Color + FAF | 2 | 3.019 | 0.221 |
| Control | RNFL | Color + NIR | 2 | 9.657 | 0.008 |
| Control | RNFL | Color + RNFL | 1 | 3.799 | 0.051 |
| Control | RNFL | All Modalities | 1 | 23.13 | 0 |
| Control | Color + FAF | Color + NIR | 2 | 2.606 | 0.272 |
| Control | Color + FAF | Color + RNFL | 2 | 3.586 | 0.166 |
| Control | Color + FAF | All Modalities | 2 | 19.835 | 0 |
| Control | Color + NIR | Color + RNFL | 2 | 5.599 | 0.061 |
| Control | Color + NIR | All Modalities | 2 | 15.745 | 0 |
| Control | Color + RNFL | All Modalities | 1 | 8.726 | 0.003 |

*Full results of chi-square tests of independence (df = 2) comparing confidence level distributions (High/Medium/Low) between all pairs of imaging modalities within each diagnostic group. Bonferroni correction applied within each diagnosis (ODE: 28 comparisons, α = .0018; ODD: 28 comparisons, α = .0018; Control: 28 comparisons, α = .0018). Cramer's V effect sizes reported for significant associations. Shaded rows indicate statistically significant differences (adjusted p < .050). df = degrees of freedom;* *X² = chi-square statistic; p = probability value.*

**Supplementary Table S5.** Number of cases and overall percentage for interrater reliability between the two graders for ODE, ODD and Control data presented in eight variations of imaging modality.

|  | ODE | ODD | Control |
| --- | --- | --- | --- |
| Sample Size | N = 60 | N = 59 | N = 30 |
| Color | 45 (75.0%) | 36 (61.0%) | 19 (63.3%) |
| FAF | 30 (50.0%) | 38 (64.4%) | 20 (66.7%) |
| NIR | 38 (63.3%) | 35 (59.3%) | 17 (56.7%) |
| RNFL | 37 (61.7%) | 39 (66.1%) | 27 (90.0%) |
| Color + FAF | 37 (61.7%) | 40 (67.8%) | 20 (66.7%) |
| Color + NIR | 38 (63.3%) | 46 (78.0%) | 22 (73.3%) |
| Color + RNFL | 51 (85.0%) | 38 (64.4%) | 26 (86.7%) |
| All Modalities | 55 (91.7%) | 52 (88.1%) | 30 (100.0%) |
